# Cellular senescence in skin‐related research: Targeted signaling pathways and naturally occurring therapeutic agents

**DOI:** 10.1111/acel.13845

**Published:** 2023-04-11

**Authors:** Aleksandra Dańczak‐Pazdrowska, Justyna Gornowicz‐Porowska, Adriana Polańska, Violetta Krajka‐Kuźniak, Maciej Stawny, Aleksandra Gostyńska, Błażej Rubiś, Sarah Nourredine, Sarah Ashiqueali, Augusto Schneider, Tamara Tchkonia, Saranya P. Wyles, James L. Kirkland, Michal M. Masternak

**Affiliations:** ^1^ Department of Dermatology Poznan University of Medical Sciences Poznan Poland; ^2^ Department and Division of Practical Cosmetology and Skin Diseases Prophylaxis Poznan University of Medical Sciences Poznan Poland; ^3^ Department of Dermatology and Venereology Poznan University of Medical Sciences Poznan Poland; ^4^ Department of Pharmaceutical Biochemistry Poznan University of Medical Sciences Poznan Poland; ^5^ Department of Pharmaceutical Chemistry Poznan University of Medical Sciences Poznan Poland; ^6^ Department of Clinical Chemistry and Molecular Diagnostics Poznan University of Medical Sciences Poznan Poland; ^7^ Burnett School of Biomedical Sciences College of Medicine, University of Central Florida Orlando Florida USA; ^8^ Faculdade de Nutrição Universidade Federal de Pelotas Pelotas RS Brazil; ^9^ Department of Physiology and Biomedical Engineering Mayo Clinic Rochester Minnesota USA; ^10^ Department of Dermatology Mayo Clinic Rochester Minnesota USA; ^11^ Department of Head and Neck Surgery Poznan University of Medical Sciences Poznan Poland

**Keywords:** cellular senescence, senescence‐related skin disorders, senolytic agents, senomorphic agents, skin aging

## Abstract

Despite the growing interest by researchers into cellular senescence, a hallmark of cellular aging, its role in human skin remains equivocal. The skin is the largest and most accessible human organ, reacting to the external and internal environment. Hence, it is an organ of choice to investigate cellular senescence and to target root‐cause aging processes using senolytic and senomorphic agents, including naturally occurring plant‐based derivatives. This review presents different aspects of skin cellular senescence, from physiology to pathology and signaling pathways. Cellular senescence can have both beneficial and detrimental effects on the skin, indicating that both prosenescent and antisenescent therapies may be desirable, based on the context. Knowledge of molecular mechanisms involved in skin cellular senescence may provide meaningful insights for developing effective therapeutics for senescence‐related skin disorders, such as wound healing and cosmetic skin aging changes.

## Graphical abstract

Aging and external stressors including exposure to UV light promote senescence of keratinocytes accelerating aging process. Development of effective plant‐based senotherapeutics represent novel approaches for senescent‐related skin disorders and can be used in cosmetic skin changes related to aging.

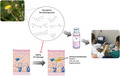

**Abbreviations**
DEJ: dermo‐epidermal junction
ECM: extracellular matrix
IGF: insulin‐like growth factor
MAPK: mitogen‐activated protein kinases
ROS: reactive oxygen species
SA‐β‐eGal: senescence‐associated β‐galactosidase
SASP: senescence‐associated secretory phenotype
SCAPs: senescent cell antiapoptotic pathways
SC: stratum corneum
TEWL: transepidermal water loss
UVR: ultraviolet (UV) radiation

## 1 INTRODUCTION

Over the past decade, cellular senescence, an essentially irreversible cell cycle state that can increase with aging, has garnered attention from researchers involved in biomedical and pharmaceutical studies. This pleiotropic cell fate is discussed in physiological conditions as well as in age‐related chronic disorders (e.g., osteoporosis, neurodegenerative diseases, cancers, idiopathic pulmonary fibrosis, metabolic dysfunction–type 2 diabetes, and obesity) (Kaur & Farr, 2020). However, the role of cellular senescence in human skin conditions remains equivocal. This article aims to review advances and summarize different aspects concerning skin cellular senescence, from physiology to pathology and signaling pathways including the role of miRNA. Knowledge about cellular mechanisms and signaling pathways involved in skin cellular senescence could facilitate development of novel root‐cause targeted therapeutic approaches, including naturally occurring plant‐derived agents, as topical skin applications.

## 2 FUNCTIONAL AND PHYSIOLOGICAL CHARACTERISTICS OF SKIN SENESCENCE

**FIGURE 1:**
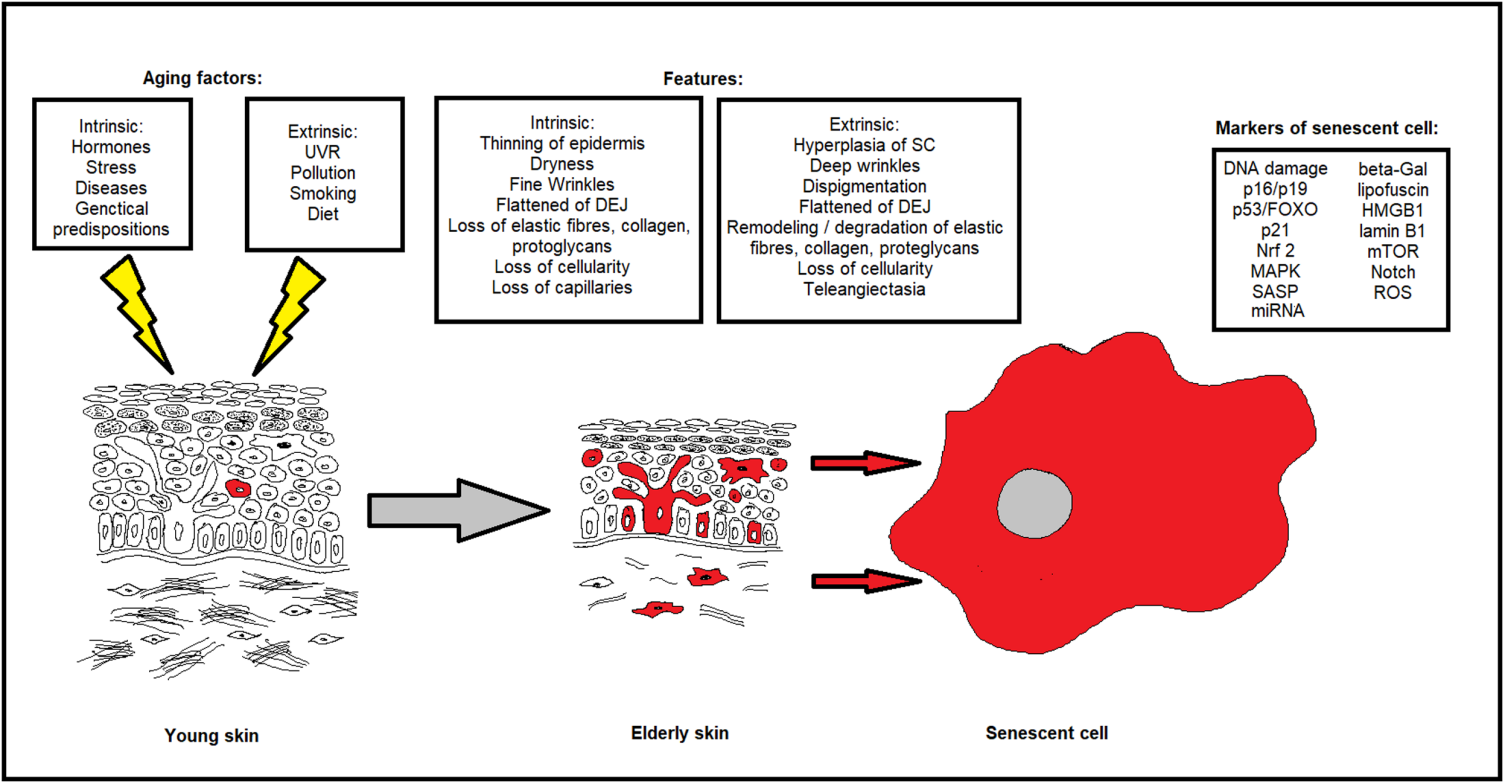
Structural differences between young (left panel) and aged, elderly skin (middle panel). Senescent cells (red) accumulate in aged skin. Right panel: Senescent cells are characterized by upregulation of p21 and p16, secretion of the senescence‐associated secretory phenotype (SASP), increased β‐galactosidase activity, translocation of HMGB1, reduced laminin B1 expression, accumulation of ROS, aberrant of the MAPK‐FOXO3, and modulation of the Nrf2, mTOR and Notch pathways, and DNA damage. DEJ, dermo‐epidermal junction; SC, stratum corneum.

Skin is characterized by complex architecture that comprises highly specialized cell types with different proliferative capacities that are adapted to perform distinct functions. The skin plays a significant role in protection related to the epidermis and its stratum corneum (SC), which regulate water loss from the body (Transepidermal Water Loss, TEWL) and reduce the impact of environmental insults and pathogens (Chambers & Vukmanovic‐Stejic, 2020; Wilhelm et al., 1991). Skin aging is related to both intrinsic (physiological process) and extrinsic factors (exposome such as ultraviolet radiation (UVR), pollutants, or smoking), which induce progressive disturbance of the skin structure and its physiological functions, causing impaired barrier function, disrupted wound healing, increased inflammation, and enhanced susceptibility to skin cancers (Farage et al., 2008; Krutmann et al., 2014). Morphological changes of aging skin may differ among people exposed to different environmental factors. Generally, intrinsic (chronological) aging is characterized by dry skin (roughness of the skin surface) and fine wrinkles. Histologically, skin aging is characterized by thinning of the epidermis, loss of melanocytes (pale skin) and Langerhans cells, flattened dermo‐epidermal junction (DEJ) (reduced contact between epidermis and dermis), loss of fibroblasts, degradation of extracellular matrix components (including collagen, elastic fibers, oligosaccharides), and loss of capillaries (Langton & Halai, 2016; Zorina et al., 2022). Extrinsic factors can also lead to thickened epidermis (especially the SC), hypertrophic, abnormal melanocytes (pigmentary changes), accumulation of abnormal elastic fibers (elastosis), and truncated fibrillin (Trojahn et al., 2015; Zargaran et al., 2022). A significant role of the exposome is related, inter alia, to the aforementioned UVR, which leads to impaired proliferation and simultaneously upregulated p16^INK4a^, p21^CIP/Waf‐1^, and p53 in human fibroblasts, keratinocytes, and prematurely aged skin (Araviiskaia et al., 2019; Low et al., 2021; Waaijer et al., 2012). A schematic representation of skin aging focused on the role of cellular senescence is represented in Figure 1.

Understanding the mechanisms underlying skin aging, including damage to nuclear DNA, ROS generation, and mitochondrial dysfunction as well as the release of proinflammatory cytokine cell senescence‐associated secretory phenotype (SASP) components, reveals that there are multiple causal processes involved (Ho & Dreesen, 2021; Low et al., 2021; Schumacher et al., 2021). In the 1960's, Hayflick first proposed the theory of aging at the cellular level. Together with Moorhead, he found that human lung fibroblasts in culture lose their proliferative capacity and enter an essentially irreversible cell cycle arrest state, cellular senescence (Hayflick & Moorhead, 1961). The first study documenting the accumulation of senescent cells in the skin of aging baboons was presented by Jeyapalan et al. (2007). Currently, there is increasing evidence that cellular senescence is a fundamental mechanism underlying skin aging, especially in photodamaged skin, and dermal fibroblasts appear to be the most important cells involved in this process (Cavinato & Jansen‐Dürr, 2017; Ho & Dreesen, 2021; Low et al., 2021). However, senescent cells can be detected in nearly all skin compartments, including benign naevi, preneoplastic lesions, and at sites of age‐related pathologies. Being metabolically active, senescent cells can release cytokines and extracellular matrix‐modifying enzymes and other molecules that can impair the integrity and function of the skin (Acosta et al., 2013; Ho & Dreesen, 2021; Wang & Dreesen, 2018; Yoon et al., 2018).

### 2.1 Keratinocytes and melanocytes

Keratinocytes, the most abundant cell type in the epidermis, may account for relatively little senescent cell accumulation with aging, although keratinocytes are constantly impacted by various damaging factors (Ho & Dreesen, 2021; Idda et al., 2020). The subpopulations of keratinocytes in the epidermis are distinct according to their differentiation status and function. Terminally differentiated keratinocytes that undergo desquamation are less prone to accumulating damaged material, and unlike skin fibroblasts, they generally undergo apoptosis as a damage response. However, epidermal turnover slows in the elderly, and a gradual decrease of innate mechanisms may favor the accumulation of senescent cells in aged skin (Chambers & Vukmanovic‐Stejic, 2020; Ho & Dreesen, 2021). Cellular senescence markers have been detected in the epidermis within the stratum basale, spinosum, and granulosum, using senescence‐associated β‐galactosidase (SA‐β‐eGal), p21^CIP/WAF‐1^, loss of lamin B1, and increased p16^INK4a^, telomere associated foci (TAF's), HMGB1, and H2A.J (Dreesen et al., 2013; Low et al., 2021; Rübe et al., 2021; Victorelli et al., 2019). Given that p16^INK4a^ positive cells seem to be limited to the basal layer and resemble melanocytes, it appears that senescent melanocytes are more abundant than senescent keratinocytes. Some authors suggest that senescent keratinocytes in situ might have p16^INK4a^ levels below the detection threshold level (Low et al., 2021; Ressler et al., 2006). Also, the reduction of lamin B1 is a marker of senescence and for the effects of environmental insults on primary human keratinocytes in vitro and skin aging and regeneration in vivo due to UVB exposure (Wang et al., 2017).

Melanocytes produce melanin, which is photoprotective for adjacent keratinocytes. Melanocytes can express p16^INK4a^ in aging skin and lesions related to photodamaged skin and are a major population of senescent cells in the epidermis (Low et al., 2021; Victorelli et al., 2019). Melanocytes can have additional markers of cellular senescence, such as reduced HMGB1 and dysfunctional telomeres, without detectable telomere shortening. Moreover, senescent melanocytes can induce senescence through paracrine activity and provoke a decline in proliferation of surrounding cells. The paracrine activity of senescent melanocytes impairs basal keratinocyte proliferation and contributes to epidermal atrophy in 3D human epidermal equivalents, a histological hallmark of skin aging (Victorelli et al., 2019). Using this model, Victorelli et al. (2019) also examined the Bcl‐2 family and their inhibitor ABT‐737, reporting that ABT‐737 can effectively eliminate senescent melanocytes. However, this is in contrast to Kohli et al. (2022), who demonstrated in human and mouse senescent melanocytes the overexpression of antiapoptotic Bcl‐w, from the Bcl‐2 family, together with the resistance to the toxic effect of ABT‐263 or ABT‐737. Thus, further experiments are necessary to explain if senolytic efficacy depends on culture conditions. Furthermore, it should be noted that senolytics may have variable effects on different skin cells, probably because of the high heterogeneity observed across different types of senescence (Kohli et al., 2022).

### 2.2 Fibroblasts

The main contributor to skin senescence is fibroblasts, which constitute the largest population responsible for the organization of the extracellular matrix (ECM). The proliferation rate of fibroblasts in vivo is much slower than keratinocytes. Fibroblasts have lower antioxidant capacity and are less efficient in nucleotide excision repair. Indeed, senescent fibroblasts induce skin aging through their proliferation arrest and SASP, which provokes an inflammatory state, degrades the ECM, and disrupts the tissue microenvironment (Herbig & Sedivy, 2006; Low et al., 2021; Ressler et al., 2006; Wang & Dreesen, 2018). As the main component of connective tissue parenchyma, fibroblasts also contribute to whole body aging and age‐related disorders. Both in vitro and in vivo age‐dependent increases in p16^INK4a^ expression in human senescent fibroblasts have been observed (Ho & Dreesen, 2021; Low et al., 2021). Differences in morphology and function between papillary and reticular fibroblasts have been debated (Ressler et al., 2006). It has been suggested that papillary fibroblasts may be differentiated into reticular fibroblasts during aging, while senolytics may change their phenotype back into papillary cells. Additionally, human fibroblast mitochondrial dysfunction has been associated with skin senescence characterized by lower NAD/NADH ratios and a distinct SASP independent of the IL‐1 pathway (Wiley et al., 2016).

Beyond their localized effect in the dermis, fibroblasts also contribute to a functional interaction between the dermis and the epidermis during aging. The main protein responsible for keratinocyte proliferation is IGF‐1, which is secreted by dermal fibroblasts and activates the IGF‐1 receptor on keratinocytes. Lewis, et al. found that IGF‐1 from fibroblasts is essential for appropriate UVB response of keratinocytes, and its expression is reduced in senescent fibroblasts in vitro (Lewis et al., 2010).

## 3 CELLULAR SENESCENCE AND ASSOCIATED SIGNALING CASCADES

Cellular senescence is a reaction of normal cells to various types of stress. Recent studies have provided essential insights by which different stresses activate the signaling pathways leading to senescence. These studies demonstrated that a combination of different physiologic stresses acting simultaneously has a crucial impact on growing cell populations (Ben‐Porath & Weinberg, 2005). Additionally, analysis of signaling pathway activation by evaluating specific proteins and markers of proliferation, apoptosis, or the cell cycle allowed prediction of whether cells enter the senescence process.

The first reports of cellular senescence under stress appeared 40 years ago, but understanding the mechanism of this process depended on specific markers (Jeyapalan et al., 2007). Explaining the functional decline of cells, organs, and surrounding microenvironments was possible by evaluating the activation of signaling pathways, their essential proteins and kinases. Among the crucial pathways controlling the cell cycle and often used in the assessment of cellular senescence are p16^INK4a^/Rb (retinoblastoma protein) and p19^ARF^/p53/p21^CIP/WAF‐1^ (Aravinthan, 2015; Csekes & Račková, 2021, and Wang & Dreesen, 2018). Subsequent studies have shown that during senescence in a mouse model, reduced levels of the mitotic checkpoint protein BubR1 was noted, and the BubR1^H/H^ mice age prematurely. In addition, Baker et al. (2008) pointed out the opposing role of the p16 and p19 tumor suppressors. Inactivation of p16^Ink4a^ in BubR1‐insufficient mice attenuated both cellular senescence and premature aging. Conversely, p19^Arf^ inactivation stimulated senescence and aging in BubR1 mutant mice (Baker et al., 2008). Moreover, BubR1 insufficiency is considered a trigger for Cdkn2a locus activation in certain mouse tissues, with p16^Ink4a^ as an effector and p19^Arf^ as an attenuator of cellular senescence in these tissues. Another pathway required for cell cycle progression is p21^Cip1/Waf1^, which is upregulated by senescence‐inducing signals (Csekes & Račková, 2021). Furthermore, Mitogen‐Activated Protein Kinases (MAPK) are involved in triggering cellular senescence and activation of numerous soluble factors, collectively termed the SASP. Additionally, MAPKs and their upstream activators and downstream effectors are involved in physiological and pathological processes, including cancers and other age‐related diseases (Anerillas et al., 2020). In addition, the Notch pathway, which relies on ligand‐dependent activation and subsequent cleavage to liberate the Notch1 intracellular domain (N1ICD) (Kopan, 2012), has been shown to induce cellular senescence. Secondary oncogene‐induced senescence in vitro and in vivo also requires Notch, rather than the SASP alone. Moreover, Notch signaling weakens, but does not abolish, the SASP in secondary senescence (Teo et al., 2019). Furthermore, silencing of the *Nrf2* gene, the expression of which decreases with age, is associated with induction of premature senescence. Nrf2‐ARE signaling pathways are involved in cell redox balance and reduce intracellular oxidative stress damage, delaying cellular senescence and preventing age‐related diseases (Yuan et al., 2021). Recently, the role of p53, which regulates cell cycle arrest, DNA repair, and apoptosis, has been linked to inducing cellular senescence secondary to loss of p53 function and chromosomal instability (Mijit et al., 2020). Moreover, Hu et al. (2021) demonstrated that genes associated with cellular senescence such as p21 and p53 were reduced in vitro and in vivo models. Interesting is the role of Forkhead box O (FOXO), which modulates the cell cycle, apoptosis, and metabolism, and its misregulation is linked to numerous diseases, including melanoma. The FOXO4‐p53 axis and its influence on cellular senescence has suggested it might be a druggable target for age‐related diseases and morbidity (Bourgeois & Madl, 2018). These signaling pathways may provide insights for targeting transduction pathways involved in cellular senescence.

## 4 DNA DAMAGE IN SENESCENCE

Cell division is accompanied by an increased risk of mutations in replicating DNA. Increased proliferation is associated with increased risk for malignancy or at least increased abnormalities that accompany aging (Risques & Kennedy, 2018). Aging processes advance over time due to exposure to intrinsic factors (e.g., genetic factors, hormones) and extrinsic factors (e.g., UV exposure, pollution) that contribute to redox imbalance and DNA damage (Farage et al., 2008; Krutmann et al., 2014). Aging of the skin is a multifactorial process that, more than any other tissue, is affected by photodamage. This specifically refers to UV radiation (mainly UVA and UVB) that can contribute to DNA damage and cancer development (Jiang et al., 2020; Lee et al., 2020; Lin et al., 2021). Although a weak mutagen, UVA penetrates into the dermis and triggers oxidative stress and inflammation (Rünger & Kappes, 2008). UVB is more mutagenic and directly interacts with DNA to generate dipyrimidine photoproducts, leading to DNA damage during DNA replication (Bastien et al., 2013). In vitro evaluation of UVB‐exposed skin fibroblasts and keratinocytes indicated DNA damage, cell cycle arrest, and elevated cellular senescence as shown by increased SA‐β‐gal activity, p16^INK4a^, p21^CIP1/WAF1^, p53 activation, and lamin B1 downregulation (Wang & Dreesen, 2018).

Similarly, in vivo exposure to UVB led to DNA damage and lamin B1‐low senescent cells within mouse epidermis but not the dermis (Wang et al., 2017). UVB exposure led to reduction in progenitor cell numbers in the hair follicle and p21^CIP1/WAF1^ accumulation in the epidermis and hair follicle progenitor cell region (McCart et al., 2017). Another study showed that tobacco extracts applied to skin and oral fibroblasts induced cellular senescence, including premature cell cycle arrest, oxidative DNA damage, secretion of inflammatory cytokines and MMPs, and downregulation of the cell junction proteins, E‐cadherin and ZO‐1 (Coppe, Boysen, et al., 2008; Coppe, Patil, et al., 2008). Eventually, persistent DNA damage, either by impaired DNA replication, repair, or signaling, resulted in premature cellular senescence and accelerated skin aging (Carrero et al., 2016). This, in turn, may predispose to cancer (Kaur & Farr, 2020).

## 5 ROLE OF CELLULAR SENESCENCE IN SKIN PATHOLOGICAL CONDITIONS

Premature cellular senescence can underlie pathological skin conditions. However, its role is ambiguous, and it seems that a distinction needs to be made between acute and chronic senescence. It appears that the chronic accumulation of senescent cells can have a detrimental effect on skin function, health, and aging, while acute stimulation of transient senescent cells plays an important role in wound healing.

Wound healing is a complex, multistep process that involves inflammation, proliferation, and remodeling (Childs & Murthy, 2017). Accumulation of short‐lived senescent fibroblasts was observed at sites of healing cutaneous wounds (Demaria et al., 2014). Transient induction of the SASP, due to its antifibrotic and proangiogenic properties, may have a beneficial effect on wound healing by promoting skin tissue remodeling. It contributes to repair largely by inducing myofibroblast differentiation through senescence‐associated secretion of platelet‐derived growth factor AA (PDGF‐AA) (Demaria et al., 2014; Jun & Lau, 2010; Thanapaul et al., 2021; Zhang et al., 2019).

In contrast to acute wound healing, chronic, nonhealing ulcers (e.g., murine model of diabetes, venous ulcers, radiation ulcers) are characterized by higher levels of senescence (Stanley & Osler, 2001; Wang, Liu, et al., 2020; Wang, Wang, et al., 2020; Wilkinson et al., 2019). Clearance of senescent cells with senolytics (i.e., dasatinib plus quercetin) has been shown to mitigate radiation ulcers (Wang, Liu, et al., 2020; Wang, Wang, et al., 2020). Additionally, it is well known that the regenerative capacity of the skin declines with age in conjunction with increased accumulation of senescent cells (Thanapaul et al., 2021). Delayed wound healing was reported in young 8‐week‐old mice after the subcutaneous transfer of irradiated fibroblasts. In this case, the kinetics of wound healing were similar to those observed in naturally aging 2‐year‐old mice (Thanapaul et al., 2022). On the other hand, Chia, et al. reported different senescent responses in younger (30.2 ± 1.3 years) vs. older (75.6 ± 1.8 years) healthy subjects in punch biopsy wounds. The second biopsy, taken for analysis, was performed several days after the first one. Induction of p21 and p53 was observed during healing in younger but not older skin (Chia et al., 2021). Therefore, transient appearance of senescent cells may be needed for proper healing of acute wounds, but their chronic presence delays healing (Wilkinson & Hardman, 2020). Moreover, senescent fibroblasts have also been found in keloid scars–lesions that result from abnormal wound healing and can be classified as benign skin tumors. It is believed that the appearance of aging is a desirable phenomenon in keloids as a mechanism potentially responsible for stopping the proliferation of fibroblasts and the progression of lesions (Varmeh et al., 2011; Wang et al., 2023).

Importantly, other skin neoplasms have been also studied in the context of cellular senescence. For example, seborrheic keratosis is one of the most common, benign forms of age‐related epidermal growth. Cells isolated from seborrheic keratotic lesions are characterized by increased p16 expression and longer survival time than peripheral skin keratinocytes (Nakamura & Nishioka, 2003). Nonmelanoma skin cancers are age‐associated skin lesions typically located on sun‐exposed skin. Cellular senescence is considered a key mechanism that protects from tumor formation, proliferation, and expansion through cell cycle arrest of premalignant cells. This has been found in actinic keratoses, a precancerous lesion that is secondary to UV irradiation, in which high levels of cellular senescence have been observed in the stromal cells of preinvasive lesions. In contrast, in invasive skin lesions such as squamous cell carcinoma, senescent stromal fibroblasts are scarce (Procopio et al., 2015). In many studies of cancers other than skin neoplasms, lower levels of cellular senescence were associated with poor survival and prognosis (Wyld et al., 2020). It has been postulated that SASP components may enhance immune surveillance of tumors and induce cellular senescence, thus inhibiting tumor expansion (Zhang et al., 2019). However, it has also been suggested that proangiogenic, proinflammatory growth factors as well as metalloproteinases released by persistent senescent cells may exert tumor‐promoting effects (Alimirah et al., 2020; Coppe et al., 2006; Coppe, Boysen, et al., 2008; Coppe, Patil, et al., 2008). Recently, an animal model of squamous cell carcinoma (*p16‐3MR* transgenic mice) provided evidence that cellular senescence promotes skin carcinogenesis through p38^MAPK^ and p44/p42^MAPK^ signaling (Alimirah et al., 2020). In contrast to squamous cell carcinoma, cutaneous melanoma is less common. However, it is one of the most aggressive skin neoplasms. Mutations in oncogenes such as BRAF, NRAS, and NF1 are involved in melanoma pathogenesis. These mutations, BRAF and NRAS, were also found in acquired or congenital naevi. It appears that melanoma can arise from pre‐existing naevi (mainly those located on the nonchronically sun damaged skin, e.g., on the torso). Nonetheless, oncogenic mutations appear insufficient to cause malignant transformation (Damsky & Bosenberg, 2017; Leclerc et al., 2017). Naevi cells are characterized by senescence markers such as SA‐β‐Gal, phosphatase, and tensin homolog (PTEN) or p16^INK4a^ expression, resulting in growth arrest (Gray‐Schopfer et al., 2006; Leclerc et al., 2017; MacKenzie Ross et al., 2013; Sung & Chang, 2022). It has also been reported that dysplastic naevi and early melanomas express less senescent markers (Gray‐Schopfer et al., 2006; MacKenzie Ross et al., 2013). Therefore, senescence evasion is an important part of melanoma pathogenesis (Leclerc et al., 2017, Sung & Chang, 2022). To sum up, the role of cellular senescence in carcinogenesis is complex, and further research is needed to elucidate it.

Premature skin aging has been noted in chronic acquired pigmentation disorders, including hyperpigmented (e.g., solar lentigo or melasma) and hypopigmented (e.g., vitiligo) lesions (Bellei et al., 2013; Kim et al., 2019; Yoon et al., 2018). Solar lentigo is an age‐related pigmentation that accumulates over time secondary to sun exposure. Increased senescent fibroblasts have been found in hyperpigmented skin compared to the surrounding tissue (Yoon et al., 2018). Melasma is a multifactorial disease that can be exacerbated by skin photoaging. Senescent fibroblasts and keratinocytes have been observed in melasma‐affected skin compared to perilesional areas (Kim et al., 2019; Lee & Kim, 2021). Clearing senescent cells using radiofrequency resulted in skin lightening in hyperpigmentation disorders (Kim et al., 2019; Lee & Kim, 2021; Yoon et al., 2018). Unlike melasma, vitiligo is an autoimmune disease that can affect people of any age. In these patients, senescent cells, including keratinocytes, melanocytes, and fibroblasts, were detected in lesional and nonlesional skin (Bellei et al., 2013; Lee et al., 2020; Rani et al., 2017).

There is also evidence in the literature for the role of senescence in systemic sclerosis, which is a systemic autoimmune disease in which fibrosis of the skin is one of the main clinical manifestations (Martyanov et al., 2017). Moreover, a recent single‐arm clinical trial suggested the usefulness of senolytics in the treatment of patients with SSc (Martyanov et al., 2017, 2019). In this study, 31 patients with SSc received dasatinib for 169 days. Skin biopsies before and after treatment were taken from 12 of them. The mean change in modified Rodnan Skin Score from baseline to Day 169 (end of therapy) was not statistically significant, but was −7.6 ± 4.7 (*p* = 0.0002) during the follow‐up (Day 365). The analysis of skin biopsies revealed a significantly attenuated SASP gene signature from baseline in responders versus nonresponders. Interestingly, responders were characterized by a significantly higher level of SASP factors before the therapy compared to nonresponders. In addition, it has been shown that navitoclax (ABT‐263), which exerts a proapoptotic effect, can reverse the established fibrosis in the bleomycin‐induced mouse model of scleroderma (Lagares et al., 2017).

Beyond autoimmune diseases such as aforementioned vitiligo and systemic sclerosis, a role for immunosenescence in bullous pemphigoid, cutaneous lupus erythematosus, and psoriasis has been postulated (Deotto et al., 2022; Mercurio et al., 2020; Wang et al., 2022).

Hair disorders can also be affected by senescent pathways. In an experimental model of age‐related hair loss, hair follicle dermal stem cells exhibited features of senescent cells (overexpression of p16^INK4a^ and SA‐β‐Gal). It has been suggested that this is due to dysregulation of the AP1‐Cyr61 pathway (Shin et al., 2020). Additionally, increased SASP components were detected in the mesenchymal niche of the hair follicle (Shin et al., 2020). Moreover, clearing senescent cells by a possible senolytic, FOXO4‐DRI, reduced hair loss in progeroid aging mice (Baar et al., 2017). Androgenetic alopecia is the most common type of baldness caused by multiple pathways, including heritable mechanisms. Symptom onset can begin in the teenage years. Hair follicle dermal papillary cells from the male balding scalp showed the loss of proliferative capacity. This phenomenon was associated with increased SA‐β‐Gal and p16^INK4a^/pRb expression (Bahta et al., 2008). Moreover, knocking out p16^INK4a^ promoted faster growth of hair follicle dermal papillary cells and an increase in the number of cells in the G1 phase (Cheng et al., 2017).

Given that not all of these diseases are age‐related and some may appear at any age, we suggest the term “senescence‐related skin diseases” to encompass the broader spectrum of disorders associated with pleiotropic senescence.

## 6 PLANT‐DERIVED BIOCOMPOUNDS AND SKIN CELLULAR SENESCENCE

Senescent cells are resistant to apoptosis because of upregulation of prosurvival pathways, called senescent cell antiapoptotic pathways (SCAPs) (Chaib et al., 2022; Zhu et al., 2015). Senomorphics (senostatics) suppress senescence or their secretory phenotype without inducing apoptosis, hence providing a senotherapeutic effect distinct from senolytics (Pils et al., 2021; Woo et al., 2021; Yousefzadeh et al., 2018).

Skin cellular senescence can be suppressed with bioactive natural products and medicinal plants. The phytochemical molecules with antisenescent activity extracted from medicinal plants have been reported as safe therapeutic targets for senescence‐related skin therapy. It can be speculated that natural products may have a decelerating effect compared to modern pharmaceutical drugs, as they may work slowly but have a cumulative effect (Mohd Zaid et al., 2022).

Several plant‐derived compounds have been examined in vitro and in vivo studies of skin cellular senescence. For example, polyphenols of plant origin can exert senomorphic properties (Bian et al., 2020; Csekes & Račková, 2021; Domaszewska‐Szostek et al., 2021; Sharma & Padwad, 2020; Lämmermann et al., 2018; Wu et al., 2021; Xian et al., 2021). Based on the number of phenolic groups and structural elements, polyphenols can be classified into four chemical classes: flavonoids, stilbenes, lignans, and phenolic acids (Sharma & Padwad, 2020). There is growing evidence that especially flavonols, substances in fruits and vegetables, can target cellular pathways crucial for clearing senescent cells and reducing the SASP (Csekes & Račková, 2021; Perrott et al., 2017; Zhu et al., 2017).

The mechanisms of action of natural origin senolytic or senomorphic agents are different and not fully understood. The influence of polyphenols in preventing cellular senescence is related to oxidative stress, inflammation, and autophagy. It is postulated that antioxidants from plants are substances that may protect cells from damage by preserving stemness and reducing cellular senescence (Petruk et al., 2018). However, the senolytic effect of antioxidants depends on their dose and target cell type (Kornienko et al., 2019). Thus, high doses of antioxidants can cause DNA damage and induce premature senescence when applied to proliferating cells. Beyond their antioxidant capacity, polyphenols can interact with proteins involved in cell metabolism, proliferation, inflammation, and growth, leading to the elimination of senescent cells and neutralization of their SASP. Medicinal plants and active chemical compounds that may contribute to skin cellular senescence prevention are shown in Table 1.

**TABLE 1:** Bioactive chemicals compounds of plant origin with potential action against skin senescence.

Active chemical compound	Plant source	Chemical formula	Mechanism	Reference
Quercetin	Onions, grapes, berries, cherries, broccoli, and citrus fruits	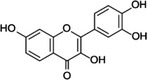	Enhance MAPK activity with nonapoptotic cell death Decrease number of stress‐induced SC Suppression of senescence‐associated proinflammatory response (decreased levels of IL‐8 and IFN‐β, senostatic action)	Lewinska et al. (2020), Hwang et al. (2018), Zhu et al. (2015)
Fisetin (5‐Desoxyquercetin)	Apples, persimmon, grapes, onions, cucumbers, and strawberries	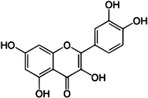	Inhibition of PI3K/AKT/mTOR pathway, topoisomerase, and senescence‐associated proinflammatory cytokines (TNFα, IL‐6)	Yousefzadeh et al. (2018), Zhu et al. (2017)
Piperlongumine	A variety of species in the genus *Piper*	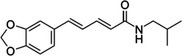	Eliminate SC through an ROS‐independent mechanism Targets Bcl‐2 family	Wang et al. (2016)
Curcumin and curcumin analog‐EF24	*Curcuma longa*, mango ginger, curry powder	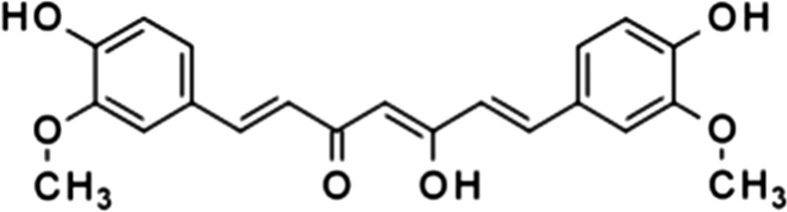	Selectively eliminate SC by inducing SC apoptosis	Li, He, et al. (2019), Li, Qin, et al. (2019)
Silymarin	*Silybum marianum*, grapes, beet greens, peanuts, brewer's yeast and berries	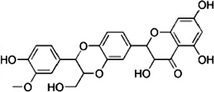	Reduce the formation of γ‐H2A.X foci, attenuate the DNA damage triggering cellular senescence Activation of the p53‐p21 pathway as well as p16	Woo et al. (2021)
Caffeine	Camellia sinensis (green/black/white tea) *Coffea* sp. (coffee) Theobroma cacao (cocoa) *Paullinia cupana* (guarana) *Ilex* sp. (Mate, guayusa, and yaupon)	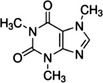	Prevent skin from oxidative stress‐induced senescence through induction of A2AR/SIRT3/MAPK‐mediated autophagy	Li et al. (2018)
Resveratrol	*Veratrum grandiflorum*, *Polygonum cupsidatum*, grapes, peanut, blueberry, bilberry, cranberry, purple grape	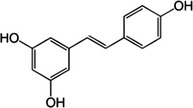	Regulation of the MAPK‐FOXO3 pathway	Ido et al. (2015)
Procyanidin C1	Grapeseed extract			Xu et al. (2021)

Abbreviations: ROS, reactive oxygen species; SC, senescent cells.

## 7 FUTURE PERSPECTIVES

Based on their phytochemical activity on molecular pathways, medicinal plants are a possibility for the protection and limitation of cellular senescence. Our knowledge is limited mostly to in vitro studies on plant‐derived compounds, with a few in vivo models showing their antisenescence activities. Further mechanistic clinical trials are needed to establish bioactive compounds’ safety, dosing, and clinical efficacy.

Interestingly, a chemoherbal combination in antisenescence approaches is suggested, such as dasatinib + quercetin (D + Q) tested in several preclinical aging‐related disease models (Chaib et al., 2022; Saccon et al., 2021; Zhu et al., 2015). In this approach, bioactive compounds from medicinal plants are administered with synthetic drugs, which appears to be feasible and effective for eliminating senescent cells from humans (Hickson et al., 2020).

Significant challenges for developing skin therapies based on plant‐derived substances are to understand their bioavailability, the best delivery systems, and determine doses of compounds for achieving optimal effectiveness with minimal side effects. The source of biocompounds can influence outcomes; therefore, standardization to achieve a consistent metabolic profile may be required (Sharma & Padwad, 2020). Additionally, synthetic senomorphics may be utilized such as metformin or rapamycin (Chung et al., 2019; Kim et al., 2022). It should be noted that bacteria such as *Lactococcus lactis* may prevent senescence processes in skin (Gervason et al., 2019). It has been reported that hydrophobic extracts of the bacterium *Sphingomonas* extract significantly reduce cellular senescence, with decreases in SA‐β‐Gal, p16, and p21 in a human skin equivalent model (Gervason et al., 2019).

Through its multifunctional and multitarget action, cellular senescence may play both beneficial (tumor suppressing, wound healing, tissue remodeling in some physiological situations, i.e., embryonic development) and detrimental roles (tumor promotion, age‐related disorders, obesity, diabetes) (Chaib et al., 2022; Tominaga, 2015). Thus, increasing evidence indicates that both prosenescent and antisenescent therapies may be desirable. Indeed, radiation and alkylating or anthracycline chemotherapy regimens induce senescence as well as apoptosis and necrosis of cancer cells. Hence, a “1:2 punch” approach comprising chemotherapy/ radiation followed by senolytics has been proposed as a potential therapeutic approach for cancer treatment clinical trials (Guida et al., 2021; Prasanna et al., 2021; Rahman et al., 2022).

In general, systemic administration of oral senolytics for senescence‐associated diseases is preferred due to the spread of senescent cells across different organs throughout the whole organism (Iske et al., 2020; Wang, Liu, et al., 2020; Wang, Wang, et al., 2020). The skin remains a possible exception where local or topical applications may be effective (Chaib et al., 2022). Therefore, future studies examining topical application of natural biocompounds as a new strategy for preventing age‐associated dermatoses are warranted. Since senescent skin cells disrupt tissue architecture through affecting the normal skin structure and integrity, it is possible that senolytic topical application could also be used for skin rejuvenation (Kohli et al., 2022).

## 8 CONCLUSIONS

Multiple roles for cellular senescence in dermatological conditions have been reported. Given that the skin is a large and accessible organ and reacts to the external and internal environments, skin provides an attractive target for investigating cellular senescence as well as the action of senolytic or senomorphic substances. This affords the opportunity to improve therapeutic approaches and discover novel antisenescence agents, especially those derived from natural sources. Innovative tools for targeting senescence pathways that have been tested and validated in clinical trials will allow personalized management of aging skin and senescence‐related disorders.

## AUTHOR CONTRIBUTIONS

ADP involved in investigation, writing, review and editing, and supervision. JGP involved in writing, review and editing, and visualization. AP involved in writing, review and editing, and visualization. MS involved in writing, review and editing, and visualization. VKK involved in writing, review and editing, and visualization. AG involved in writing, review and editing, and visualization. BR involved in writing, review and editing, and visualization. SN involved in writing, review and editing, and visualization. SA involved in writing, review and editing, and visualization. AS involved in writing, review and editing, and visualization. TT involved in writing, review and editing, and visualization. SPW involved in writing, review and editing, and visualization. JLK involved in writing, review and editing, and visualization. MMM involved in writing, review and editing, supervision, and funding acquisition.

## ACKNOWLEDGEMENT

This work was supported by by the European Union's Horizon 2020 research and innovation programme under the Marie Skłodowska‐Curie grant agreement No 778051 and the Ministry of Science and Higher Education of Poland fund for supporting internationally cofinanced projects in 2018–2022 (agreement No 3899/H2020/2018/2), the National Institute on Aging of the National Institutes of Health R15 AG059190 (M.M), R56 AG074499 (MM), R37AG013925 (JLK), and R33AG061456 (JLK), the Connor Fund (JLK), Robert J. and Theresa W. Ryan (JLK), and the Noaber Foundation (JLK) and and CNPq for (AS).

## CONFLICT OF INTEREST STATEMENT

TT and JLK have a financial interest related to this research, including patents and pending patents covering senolytic drugs and their uses that are held by Mayo Clinic. This research has been reviewed by the Mayo Clinic Conflict of Interest Review Board and was conducted in compliance with Mayo Clinic conflict of interest policies. All other authors declare no conflict of interest.

## Data Availability

Not available.
